# Increased Arterial Responsiveness to Angiotensin II in Mice Conceived by Assisted Reproductive Technologies

**DOI:** 10.3390/ijms232113357

**Published:** 2022-11-01

**Authors:** Theo Arthur Meister, Rodrigo Soria, Afzal Dogar, Franz H. Messerli, Ariane Paoloni-Giacobino, Ludwig Stenz, Urs Scherrer, Claudio Sartori, Emrush Rexhaj

**Affiliations:** 1 Department of Cardiology and Biomedical Research, Inselspital Bern University Hospital, 3010 Bern, Switzerland; 2 Tropic Biosciences Ltd., Norwich Research Park Innovation Centre, Norwich NR4 7GJ, UK; 3 Department of Genetic Medicine and Development, University of Geneva, 1205 Geneva, Switzerland; 4 Department of Internal Medicine, Lausanne University Hospital (CHUV), Faculty of Biology and Medicine, University of Lausanne, 1015 Lausanne, Switzerland

**Keywords:** assisted reproductive technologies, hypertension, DNA methylation, angiotensin II receptors

## Abstract

Since the first report in 1978, the number of individuals conceived by Assisted Reproductive Technologies (ART) has grown incessantly. In parallel, with the recent emergence of possible underlying mechanisms of ART-induced epigenetic changes in the renin-angiotensin system, the cardiovascular repercussions of ART in mice and human offspring (including arterial hypertension, vascular dysfunction, and cardiac remodeling) have become increasingly recognized. Here, we hypothesized that ART may increase arterial responsiveness to angiotensin II (ANG II) by epigenetically modifying the expression of its receptors. To test this hypothesis, we assessed the vasoconstrictor responsiveness to ANG II in isolated aortas from ART and control mice. We also examined ANG II receptor (ATR) type 1 and 2 expression and the promoter methylation of the *At1aR*, *At1bR* and *At2R* genes. We found that the vasoconstrictor response to ANG II was markedly increased in ART mice compared to controls. This exaggerated vasoconstrictor responsiveness in ART mice correlated with a significant increase in the ANG II receptor (ATR) type 1 to ATR type 2 protein expression ratio in the aorta; this was mainly driven by an increase in AT1R expression, and by hypomethylation of two CpG sites located in the *At1bR* gene promoter leading to increased transcription of the gene. We conclude that in mice, ART increase the vasoconstrictor response to ANG II in the aorta by epigenetically causing an imbalance between the expression of vasoconstrictor (AT1R) and vasodilator (AT2R) ANG II receptors. Unbalanced expression of AT1R and AT2R receptors seems to be a novel mechanism contributing to ART-induced arterial hypertension in mice.

## 1. Introduction

More than 8 million apparently healthy children have been conceived worldwide with assisted reproductive technologies (ART); it has been estimated that by the year 2100, ART individuals will account for one to three percent of the global population [1]. Recently, however, concerns have been raised about the possible long-term cardiovascular and/or metabolic repercussions of ART. For example, we and others previously observed endothelial dysfunction, vascular remodeling and increased arterial blood pressure in ART children [2,3,4,5]. More recently, in a follow-up study of ART children, we showed that endothelial dysfunction persisted into young adulthood and translated into the development of clinical hypertension [6].

The mechanisms underpinning ART-induced alteration of the cardiovascular phenotype are incompletely understood. Studies in humans are limited by socio-demographic confounders, heterogeneity of the reproductive technologies used and small sample sizes. Mouse models of ART display similar vascular dysfunction and elevation of systemic blood pressure to human models [7,8] and may be useful to answer pertinent mechanistic questions. In line with this concept, we previously showed that hypermethylation of the endothelial nitric oxide synthase (*eNOS*) gene promoter alters the expression and function of this gene; thereby, contributing to increased blood pressure in ART mice.

Recently, the effect of ART on the angiotensin II receptors (AT1R and AT2R) has gained attention. Wang and colleagues found increased cardiac AT1R mRNA expression in ART mice starting the third week after birth and persisting for as long as 1.5 years [9]. Moreover, Zhang and colleagues reported partial hypomethylation of the *At1bR* gene in the umbilical vein of human ART [10]. However, to the best of our knowledge, ANG II sensitivity, ATR expression and gene promoter methylation have never been studied in the more relevant systemic arteries of ART offspring.

We therefore assessed the vasoconstrictor responsiveness to ANG II in the aorta of ART and control mice. To provide insight into underpinning mechanisms, we measured ATR protein and gene expression and assessed DNA methylation levels of its promoter regions. To confirm ART-induced alteration of the cardiovascular phenotype in our experimental model, we measured arterial blood pressure in ART and control animals.

## 2. Results

Litter size and body weight after birth were comparable in the two groups. ART altered the cardiovascular phenotype as expected, evidenced by an increase in mean arterial blood pressure (121.8 ± 7.3 vs. 114.6 ± 4.5 mmHg ART vs. Ctrl, *p* = 0.02).

### 2.1. Increased ANG II Sensitivity Leads to Exaggerated Aortic Vasoconstriction in ART Mice

**Figure 1:**
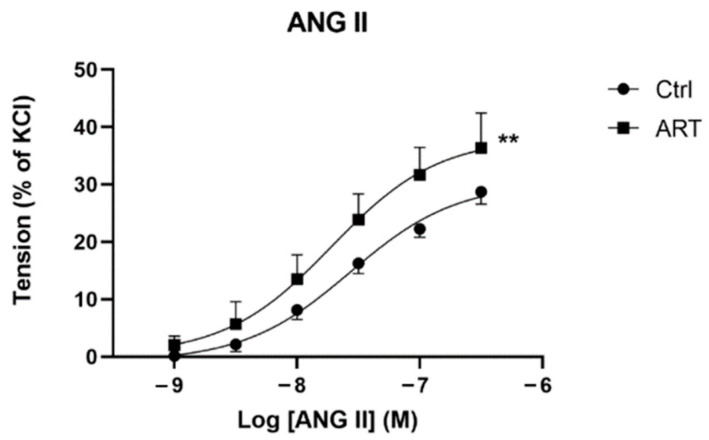
Vasoconstrictor response of aortic rings to ANG II in presence of L-NNA, measured with wire myography and expressed in % of maximal KCl contraction (*n* ≥ 6/group). Values are the Means ± SEM. Data were analyzed by two-way ANOVA. ** *p* < 0.01, ART vs. Ctrl.

The vasoconstrictive response to increasing doses of ANG II was significantly higher in aortic rings of ART compared to control mice (maximal response at 1 × 10^−6.5^ mmol/l: 36.4 ± 6.3 vs. 28.8 ± 5.7% of maximal KCl contraction, *p* < 0.01) (Figure 1). ANG II EC50 was also higher in controls compared to ART, indicating an increased sensitivity to ANG II in ART mice compared to controls (EC50 1.903 × 10^−8^ vs. 2.664 × 10^−8^, ART vs. Ctrl). We noted that the maximal KCl contraction (100 mmol/l) was comparable in the two groups (16.8 ± 3.8 vs. 14.8 ± 4.3 mN, ART vs. Ctrl, *p* = 0.4), thus excluding any changes in arterial muscular smooth cell density, function, or viability as potential confounding underpinning mechanisms for the difference in ANG II responsiveness between groups.

### 2.2. AT1R to AT2R Ratio in the Aorta Is Increased in ART Mice

**Figure 2:**
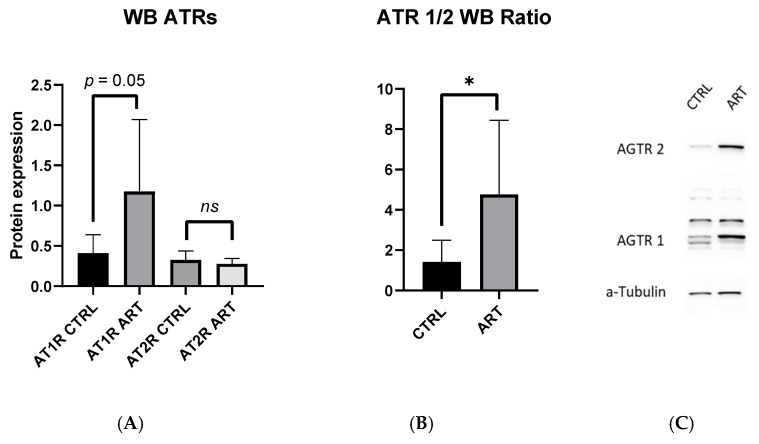
(**A**) AT1R and AT2R protein expression and (**B**) AT1R/AT2R ratio in aorta measured by Western blotting (*n* ≥ 7/group). (**C**) A representative western blot image. Values are mean ± SE. * *p* < 0.05, ART vs. Ctrl. ns = not significant.

Western blot analysis of the aorta revealed similar levels of AT1R vs. AT2R protein expression in the control (0.41 ± 0.23 vs. 0.34 ± 0.11 AT1R-CTRL vs. AT2R-CTRL, *p* = 0.4) (Figure 2A). However, the AT1R to AT2R protein expression ratio was roughly threefold greater in ART compared to the control mice (4.76 ± 3.68 vs. 1.42 ± 1.08 ART vs. Ctrl; *p* = 0.04) (Figure 2B). This imbalance of AT1R to AT2R expression was mainly driven by an almost significant increase in AT1R expression in ART compared to the control (AT1R 1.18 ± 0.89 vs. 0.41 ± 0.23, ART vs. Ctrl; *p* = 0.05). There were no statistical differences in the AT2R expression between both groups (AT2R 0.28 ± 0.07 vs. 0.33 ± 011, ART vs. Ctrl; *p* = 0.3) (Figure 2A).

### 2.3. Increased Expression of the AT1bR Gene Is Correlated with Hypomethylation of Its Promoter in ART Mice

**Figure 3:**
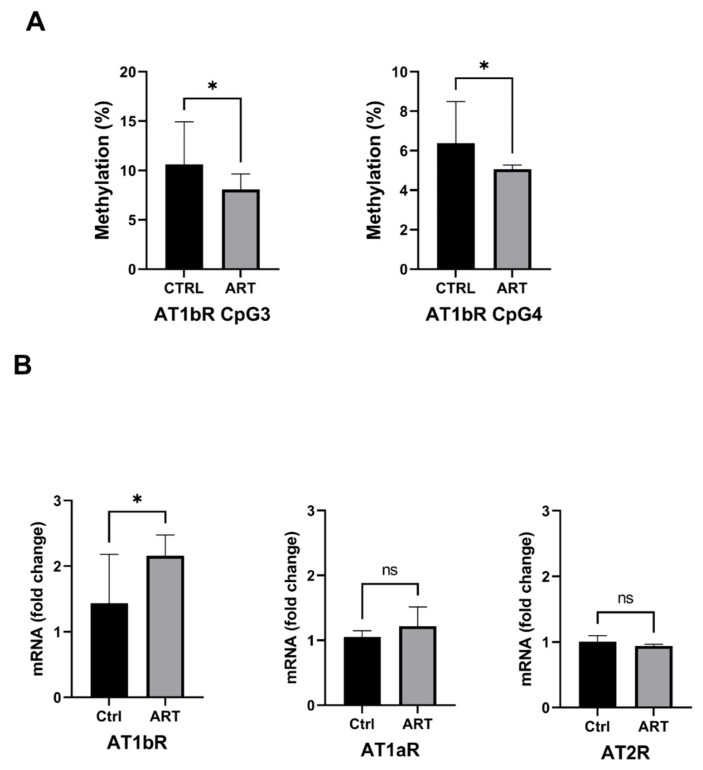
(**A**) *At1bR* gene promoter DNA methylation level determined by bisulfite pyrosequencing. (**B**) ATRs mRNA quantification in aorta by RT-qPCR in (*n* ≥ 10/group). Values are mean ± SE. * *p* < 0.05, ART vs. Ctrl. ns = not significant.

In ART mice, we found significant hypomethylation at two CpGs (−43; −63) situated just before the TATA box in the promoter region of the *At1bR* gene (CpG3: 8.1 ± 1.6 vs. 11 ± 4.3 (% of Methylation) ART vs. Ctrl; *p* = 0.04; CpG4: 5.1 ± 0.8 vs. 6.4 ± 2.1 (% of Methylation) ART vs. Ctrl; *p* = 0.03) (Figure 3A). These changes in DNA methylation correlated with a 40% increase in *At1bR* gene transcription assessed by RT-qPCR in ART compared to control mice (1.37 ± 0.35 vs. 1.01 ± 0.16 (fold to control) ART vs. Ctrl, *p* = 0.004) (Figure 3B). The methylation of the CpGs situated in the promoter regions of *At1aR* and *At2R* genes was similar in ART and controls, as was the expression of these two receptor subtypes (Figure 3B and Figure S1).

## 3. Discussion

Evidence is accumulating showing that ART alters the cardiovascular phenotype and predisposes to premature cardiovascular disease in experimental animals and humans [11]; however, the underlying mechanisms are incompletely understood. The renin-angiotensin system (RAS) is a complex enzymatic cascade culminating in the production of angiotensin II by the angiotensin-converting enzyme, localized in the vascular endothelium. RAS is an important target of fetal programing, and ATR are adversely programed in offspring prenatally exposed to impaired nutrition [12], hypoxia [13] or glucocorticoids [14]. Here, we found the in vitro vasoconstrictive response of aortic rings to ANG II was markedly enhanced in ART mice. This exaggerated response was associated with an increase in ANG sensitivity and an increase in the AT1R/AT2R protein expression ratio in the aorta. This imbalance of ATR expression was mainly driven by an increase in the AT1R expression, which appears to be related, at least in part, to an epigenetic mechanism, as evidenced by hypomethylation of the *At1bR* gene promoter correlating with an increase in its expression. Hence, perturbation of the balance between vasoconstrictor and vasodilator ANG II receptors by an epigenetic mechanism may thus represent an important new mechanism underpinning ART-induced hypertension.

Here, we used an animal model of the most complicated form of assisted reproduction possibilities: implantation of a donor early embryo in a recipient mother. Therefore, *sensu stricto*, the validity of our results is restricted to embryo donor-assisted reproduction techniques; however, previous human studies have shown that offspring born after non-donor ART exhibit similar elevations in blood pressure [6,15]. Based on these observations, we speculate that a similar mechanism could also play a role in non-donor ART.

We have previously shown that ART mice displayed endothelial dysfunction and elevation of arterial blood pressure related to epigenetically induced alteration of *eNOS* gene function [8,16]. The exaggerated vasoconstrictor response to ANG II in the present studies was not related to differences in NO bioavailability in ART mice, because prior to the ANG II studies, aortic ring preparations were pretreated with a selective and irreversible eNOS inhibitor.

We are well aware of existing differences between AT1R in rodents and AT1R in humans; in contrast to humans, rodents express two different AT1Rs (AT1aR and AT1bR) that have similar pharmacological properties and share 94% of their amino acid sequence [17,18]. Interestingly, in ART mice we found hypomethylation and increased transcription of the *At1bR* gene, which is the major mediator for ANG II contractile response in the abdominal aorta, and has been shown to mimic the single known AT1R in human vascular smooth muscle [19,20].

The present findings are in line with studies showing that adverse environmental exposure during the fetal and/or perinatal period alters vascular ANG II sensitivity through DNA methylation changes of ATR genes [21,22,23,24] and a related increase in the AT1R to AT2R ratio [21,22]. Most interestingly, the epigenetic changes in *At1bR* gene expression in the aortas of adult ART mice for the present studies were similar to those observed in the hearts of adult ART mice [9]. In our previous studies, we found that ART were associated with impaired mesenteric endothelial function caused by a reduction in eNOS; however, nitroprusside-induced vasodilation was normal [8,16]. We concluded that the arterial muscular wall or the VSMCs function were not significantly different between ART vs. Ctrl. Similarly in this study, the maximal KCl contraction was similar in the two groups. All taken together, this reduces the likelihood that the observed DNAm differences in aortic AT1bRs may be caused by changes in arterial muscular smooth cell density, function or viability.

How ART interfere with vascular regulatory epigenetic mechanisms is incompletely understood. Culture conditions during in vitro development are suboptimal and imperfectly mimic the conditions during natural conception [25], possibly interfering with epigenetically mediated gene expression in the vasculature. In line with this concept, the addition of melatonin to culture media prevented normalized *eNOS* gene promoter methylation and function and prevented endothelial dysfunction and arterial hypertension in ART mice [16].

Finally, AT1R receptor activation in arterial smooth muscle cells inactivates nitric oxide by interacting with radical producing systems such as the NADPH oxidase (thereby further aggravating the eNOS dysfunction-related impairment of NO bioavailability), and is thought to represent a key event in promoting [26,27] premature vascular aging and atherosclerosis [28]. In line with this concept, oral antioxidants had favorable effects on vascular function in the short-term in ART children [29].

In conclusion, we found that ART in mice potentiates the vasoconstrictor responsiveness to ANG II by epigenetically causing an imbalance between the expression of its vasoconstrictor and vasodilator receptors. Whether or not these abnormalities could be primarily prevented by changes in the culture conditions or are amenable to targeted intervention with ATR blockers or antioxidative drugs remains to be determined.

## 4. Materials and Methods

### 4.1. Study Approval

All animal protocols were approved by the Institutional Animal Care Committee of Canton Bern, Switzerland under number (BE 106/16+).

### 4.2. Animals

Wild-type (FVB and NMRI) mice were from the Charles-River Laboratory (L’Arbresle, France). Animals were fed a standard chow diet. Throughout the study, the mice were housed with lights on from 7:00 am to 7:00 pm, and access to food and water was ad libitum.

### 4.3. In Vitro Fertilization and Embryo Culture

These procedures were performed as described previously [8,16,30]. Briefly, 8- to 12-week-old female FVB mice were superovulated by IP injection of 5 IU (0.1 mL) pregnant mare serum gonadotropin (Intervet, Zürich, Switzerland), followed 50 h later by an IP injection of 5 IU (0.1 mL) of human chorionic gonadotropin (hCG; MSD Animal Health, Luzern, Switzerland). Fourteen hours after hCG injection, cumulus–oocyte complexes were recovered from oviducts in HTF supplemented with 5 mg/mL of HSA. Spermatozoa were collected from the cauda epididymis of 10- to 14-week-old FVB mice and capacitated for 60 min in HTF/HSA medium at 37 °C under a humidified atmosphere of 6% CO_2_ in air. Oocytes were inseminated 14 h after hCG with 10 × 10^−6^ spermatozoa in HTF/HSA medium for 4 h at 37 °C and 6% CO_2_. Eggs were then transferred to 25-µL drops of G1 medium (Vitrolife, Göteborg, Sweden) covered with paraffin oil. The embryo culture was conducted up to the blastocyst stage in sequential G1 and G2 (Vitrolife, Göteborg, Sweden) medium pre-equilibrated at 37 °C under the CO_2_-enriched atmosphere. Embryos were kept in the G2 medium for 48 h before the transfer to pseudopregnant females. The culture media used in these studies were largely used during ART in humans.

### 4.4. Embryo Transfer

NMRI females of at least 6 weeks of age were placed with vasectomized males to mate 2.5 days prior to embryo transfer. The morning after mating, females were checked for the presence of a vaginal plug. On the transfer day, pseudopregnant females were anesthetized by i.p. injection of xylazine (15 mg/kg) and ketamine (100 mg/kg). Seven to ten embryos were inserted into each fallopian tube. We used outbred NMRI mice as gestational carriers, since NMRI mice are good embryo recipients, as evidenced by the fact that they are often used for commercial embryo transfer purposes [31]. Control FVB mice were generated by normal mating of FVB mice. The 12-week-old male ART and control mice were used for in vivo blood pressure measurements and aortic in vitro and molecular studies described below. Only males were studied to avoid sex-related changes in ATR DNA methylation [21].

### 4.5. Arterial Blood Pressure

Arterial blood pressure was recorded continuously in awake mice, as described previously [8]. Briefly, a fluid-filled PE-10 tubing connected to a pressure transducer was inserted into the carotid artery under isoflurane anesthesia and tunneled subcutaneously to exit at the back of the neck. Mice were allowed to recover for 4–5 h before the blood pressure measurement. We have previously shown that measurements acquired using this method closely reflect blood pressure measurements obtained by 24 h telemetry [8].

### 4.6. Isolated Abdominal Aortic Ring Preparation and Vascular Contraction Experiments

In brief, 12-wk-old male offspring of ART and control mice were sacrificed (pentobarbital sodium 200 mg/kg, i.p.). After sacrifice of the animal, the abdominal aorta was dissected free of parenchyma and cut into a ring. We used the abdominal aorta rather than the thoracic aorta, because of its well-described vasoconstrictive response to ANG II in mice [19]. Particular care was taken to remove the perivascular adipose tissue that is known to interact with the vessel vasoreactivity [32]. Each ring mounted a small vessel myograph (DMT 620M; Danish Myo Technology, Aarhus, Denmark) [33]. The organ bath was filled with modified Krebs–Ringer bicarbonate solution (composition (in mM): 118.3 NaCl, 4.7 KCl, 2.5 CaCl_2_, 1.2 Mg_2_SO_4_, 1.2 KH_2_PO_4_, 25.0 NaHCO_3_, and 11.1 glucose), maintained at 37 ± 0.5 °C, and aerated with 95% O_2_ plus 5% CO_2_ (pH 7.4). The PowerLab 4/30 with LabChart software Pro version 7 (AD Instruments, Dunedin, New Zealand) were used for data acquisition and display. Aortic rings were stretched to their optimal resting tension and allowed to equilibrate for 1 h. To exclude potential confounding effects of variation in NO bioavailability between the groups on the results [8], aortic rings were pretreated with the NO synthase inhibitor Nw-nitro-L-arginine (L-NNA 10E-4 M) (Sigma-Aldrich, St. Louis, MO, USA) to irreversibly inhibit eNOS [34,35,36]. Viability and maximal vasoconstrictor response were assessed using 3 consecutive KCl (100 mmol/l) stimulations before serial concentrations of ANG II were measured [21]. Results are given in % of the maximal response to KCl.

### 4.7. Western Immunoblotting Analysis of Angiotensin II Receptor Proteins

Aortic ring preparations not previously used for myograph studies were homogenized in RIPA lysis buffer and then centrifuged at 4 °C for 10 min at 14,000 rpm, and supernatants were collected. Protein concentrations were measured using a protein assay kit (Pierce BCA Protein Assay Kit, ThermoFisher Scientific, Inc., Waltham, MA, USA). Equal amounts of protein (20 ug) were loaded in each lane of Novex 4 to 12% Tris-Glycinegel (Invitrogen, Canada Inc., Burlington, ON, Canada) for electrophoresis separation, and then transferred from the gel to a nitrocellulose membrane with an electroblotting apparatus. Membranes were incubated with 5% nonfat dry milk for 1 h to decrease nonspecific sites. Then, they were incubated overnight at 4 °C with the following primary antibodies: AT1R (1:800 dilution, AGTR1 Rabbit PA5-20812; Abcam, Cambridge, UK) and AT2R (1:5000 dilution, AGTR2 Rabbit ab92445; Abcam, Cambridge, UK). Samples were then washed, incubated with peroxidase-conjugated secondary antibody and assessed using an electrochemiluminescence detection kit (Bio-Rad image software; Hercules, CA, USA). Monoclonal mouse anti-α-Tubulin antibody (Merck T6074) was used as an internal control.

### 4.8. Angiotensin II Receptors mRNA Preparation and Quantification by Real-Time qPCR

RNA was extracted from aortic rings using the TRIzol protocol (Invitrogen, Burlington, ON, Canada). AT1aR, AT1bR and AT2R mRNA abundance was determined by real-time RT-PCR using thermal cycler block (Applied Biosystems, Waltham, MA, USA). The primers used were AT1aR (TaqMan^®^ Gene Expression Assay ID: Mm01957722_s1); AT1bR (TaqMan^®^ Gene Expression Assay ID: Mm02620758_s1); AT2R (TaqMan^®^ Gene Expression Assay ID: Mm00431727_g1) and Gapdh (TaqMan^®^ Gene Expression Assay ID: Mm99999915_g1). Real-time RT-PCR was performed in a final volume of 10 μL. Each PCR mixture consisted of primers, probes, and FastStart TaqMan^®^ Probe Master from Roche (4673409001). We used the following RTPCR protocol: 95 °C for 5 min, followed by 40 cycles of 95 °C for 5 s, 60 °C for 30 s, and 72 °C for 10 s. GAPDH was used as an internal reference. Each sample was assayed in triplicate, and the threshold cycle numbers were averaged. The mRNA of target genes was quantified using the ΔΔCT method and normalized to GAPDH mRNA levels.

### 4.9. DNA Methylation Quantitative Measurement by Pyrosequencing of the Angiotensin II Receptor Genes Promoters

DNA was extracted from aortic rings using the QIAampDNA Microkit (Qiagen, Hilden, Germany). Using the EZ Methylation Gold-Kit (Zymo Research, Irvine, CA, USA), the extracted DNA was treated with sodium bisulfite in order to convert unmethylated cytosine residues to uracil. The converted DNA was eluted in 10 μL of TE buffer (10 mM Tris-HCl, 0.1 mM EDTA, pH 7.5). A total of 2 μL of the bisulfite-treated DNA was used for subsequent PCR amplification. The PCR amplifications were performed starting from 80–140 ng of bisulfite-treated DNA. All reactions were performed with PCR reaction mixtures (total volume 25 μL) containing oligonucleotides at 0.5 mM concentration and 12.5 μL of HotStarTaq Master Mix (Qiagen, Hilden, Germany). The PCR conditions were the same for all genes tested, i.e., 94 °C for 15 min, followed by 40 cycles of 94 °C for 30 s, 55 °C for 30 s, 72 °C for 30 s and by a 72 °C 10-min final extension step. The characteristics of the amplicons and the oligonucleotides chosen were described earlier [37]. The biotinylated PCR products were purified using streptavidin-sepharose beads (Amersham, UK) and sequenced using the PSQ 96 Gold Reagent Kit (Biotage AB, Uppsala, Sweden). The primer sequencesand summary of the pyrosequencing assays are listed in the Supplementary Materials (Tables S1 and S2). Summary of the amplification with illustrative PCR image and pyro-histograms are available in the Supplementary Materials.

### 4.10. Statistics

Statistical analyses were performed using GraphPad Prism version 7.00 for Windows (GraphPad Software, La Jolla, CA, USA). Bivariate analyses were obtained using the unpaired 2-tailed Student’s *t*-test or 2-factor ANOVA. Post hoc comparisons were obtained using the Bonferroni multiple comparison test. A *p* value of less than 0.05 was considered to indicate statistical significance. Unless otherwise indicated, data are given as mean ±SD.

## 5. Conclusions

This is the first study to evaluate the effect of Assisted Reproductive Technologies (ART) on arterial Angiotensin II (ANG II) vasoconstrictor sensitivity in mice, and the potential role of epigenetic dysregulation of its vascular receptor expression in altering this response. The ever-growing population of ART individuals is at higher risk of developing hypertension, but its origins remain poorly understood. Here, we found that in mice, ART potentiates the vasoconstrictor response in the aorta to ANG II by epigenetically causing an imbalance between the expression of vasoconstrictor and vasodilator ANG II receptors.

The unbalanced expression of AT1R and AT2R receptors seems to be a new underlying mechanism contributing to ART-induced arterial hypertension in mice.

## Data Availability

Not applicable.

## Supplementary Materials

The following supporting information can be downloaded at: https://www.mdpi.com/article/10.3390/ijms232113357/s1.

## Abbreviations

ART	Assisted Reproductive Technologies
ANG II	Angiotensin II
AT1R	Angiotensin Receptor 1
AT2R	Angiotensin Receptor 2
eNOS	Endothelial Nitric Oxide Synthase
L-NNA	Nw-nitro-L-arginine
NO	Nitric Oxide
CpG	Cytosine-phosphate-guanine motifs
