# Supplementary material for: Increased Arterial Responsiveness to Angiotensin II in Mice Conceived by Assisted Reproductive Technologies
Source: Int J Mol Sci. 2022 Nov 1;23(21):13357. doi: 10.3390/ijms232113357 (PMC9654033; doi:10.3390/ijms232113357)

International Journal of
Molecular Sciences

Supplementary Materials

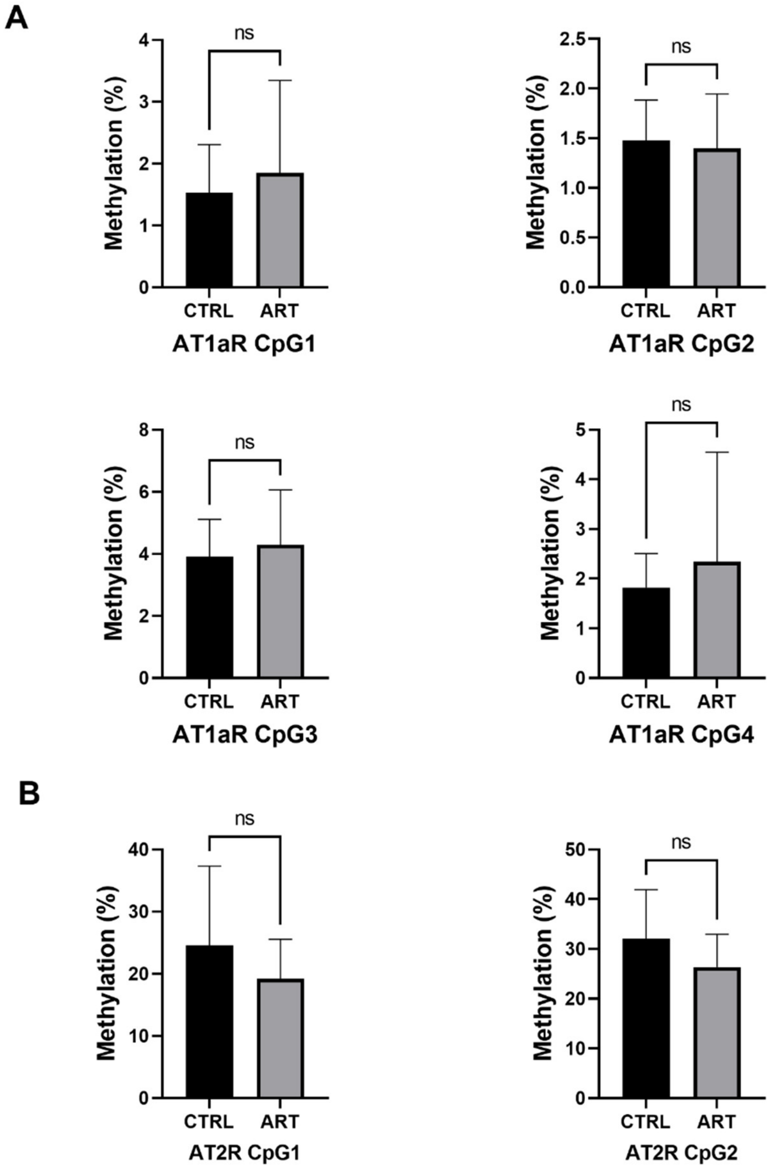

Figure S1. *At1aR* and *At2R* genes promoter DNA methylation level determined by bisulfite pyrosequencing (n ≥ 10/group). Values are mean ± SD. **A)** *At1aR*; CpG1 1.9 ±1.5 vs. 1.5 ±0.78 [% of Methylation] ART vs. Ctrl; P = 0.5. CpG2 1.4 ±0.55 vs. 1.5 ±0.41 [% of Methylation] ART vs. Ctrl; P= 0.7. CpG3 4.3 ±1.8 vs. 3.9±1.2 [% of Methylation] ART vs. Ctrl; P = 0.5. CpG4 2.3±2.2 vs. 1.8 ±0.68 [% of Methylation] ART vs. Ctrl; P= 0.4. **B)** *At2R*; CpG1 19±6.3 vs. 25 ±13 [% of Methylation] ART vs. Ctrl; P = 0.2. CpG2 26 ±6.6 vs. 32 ±9.9 [% of Methylation] ART vs. Ctrl; P= 0.08.

Supplementary material, promoter methylation in *At1aR, At1bR and At2R*

DNA methylation measured using bisulfite pyrosequencing in the promoters of *At1aR*, *At1bR* and *At2R* genes. Sequences uploaded from Mouse mm10 version. Primer designed using MethPrimer and assay designed using PyroMark q24 Advanced version 3.0.0.

Table S1. primers.

gene	Primer	Sequence (5′ to 3′)
AT1aR	AT1aR_TATA_F	GTGTAAAGTTTTAGGGTTGGAATTT
AT1aR	AT1aR_TATA_R_bio	Bio-ACCAAACAACATCATCCAATC
AT1bR	AT1bR_F	TAGAATTTGTTAAGGGAGGGGTTA
AT1bR	AT1bR_R_bio	Bio-TCCCTACTACCTAAAATCCAAACTAC
AT2R	AT2R_F	GTTGAAGGTTTTTAGTGGATAGAG
AT2R	AT2R_R_bio	Bio-CAAAATACAATTAAAATACAAAAAAA

Bio- : biotin incorporated in the 5′end of the primer.

Table S2. Resume of pyrosequencing assays.

gene	original sequences	sequence to analyse	mm10 coordinates	strand	amplicon size
**AT1aR**	GTGCAAAGCCTTAGGGTTGGAAC-<br>CTG(CG)^{1}GAGTAGCAA(CG)^{2}TCCCCCTAGGC-<br>**TATAAA-**<br>**T**ATGGAAGTGCCT(CG)^{3}CTGCTGAT(CG)^{4}*CCA*<br>*GGCAGTTGGGAGGGACTG-*<br>*GATGATGCTGTCTGGC*	GYGGAGTAG-<br>TAAYGTTTTTTTAGGTTATAAA-<br>TATGGAAGTGTTTYGTTGTT-<br>GATYGTTAGGTAGTTGGGAGGGATTGG<br>ATGATGTTGTTTGGT	chr13:30,336,361-30,336,478	+	118
**AT1bR**	CAGAATCTGTCAAGGGAGGGGTCAG-<br>GAGGGTCTTTAAGG(CG)^{1}GGG(CG)^{2}AGAAGG<br>GAAGGCAAGCCCAGACT(CG)^{3}GATCCCAA-<br>GAAACAGCAA(CG)^{4}CCCCTTTGCTG-<br>**TATAAAT**TGTAGCTGTCTC(CG)^{5}CTAGGAT-<br>TAC*AGCTGCAGGCAGCCTGGATCCCAGGCAG-*<br>*CAGGGA*	GGAGGGTTTTTAAGGYGGGGYGA-<br>GAAGGGAAGGTAAGTTTAGATTYG-<br>GATTTTAAGAAA-<br>TAGTAAYGTTTTTTTGTTGTATAAATTG<br>TAGTTGTTTTYGTTAGGATTATAGTT-<br>GTAGGTAGTTTGGATTTTAGGTAG-<br>TAGGGA	chr3:20,367,144-20,367,310	-	167
**AT2R**	GTTGAAGGCTCCCCAGTG-<br>GACAGAG(CG)^{1}AATA**TATAA**GAAGGAAAC-<br>CAGAGATCTGGTGC*AG-*<br>*TTACATCTCAGAGGCTGG(CG)^{2}ATGGAGGGAG*<br>*CT(CG)^{3}GAACTGAAAGCTTACTTCAGCCTG-*<br>*CATTTTAAGGTAAGGCAGAACAATTTACACAT*<br>*GCTTGACTAGGGAAGGGGAACATACAGCAG-*<br>*TAAAAATGTTTCTGTGTGTG(CG)^{4}TGTATGAAT*<br>*GTTTTCTTTTTCCTGTATTTTAACTGCATTTTG*	YGAATATATAAGAAGGAAATTAGA-<br>GATTTGGTGTAGTTATATTTTA-<br>GAGGTTGGYG-<br>ATGGAGGGAGTTYGGAATTGAAAGTTT<br>ATTTTAGTTT-<br>GTATTTTAAGGTAAGGTAGAA-<br>TAATTTATATATGTTT-<br>GATTAGGGAAGGG-<br>GAATATATAGTAGTAAAAATGTTTTTG<br>TGTGTGYGTG-<br>TATGAATGTTTTTTTTTTTTT-<br>GTATTTTAATTGTATTTTG	chrX:21,484,567-21,484,811	+	245

Predicted TATA boxes in bold. Primers positions underlined. Transcripts based in italic. CG sites numerated. "Y" corresponds to the C of the CpG analysed for methylation.

Resume of amplification

PCR 22mars2016 AT1aR

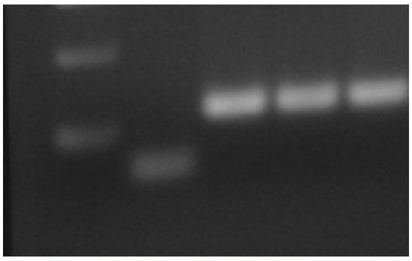

100 bp MM / BS DNA from mouse :
0ng / 50ng / 100ng / 150ng /
Assay AT1aR (118 bp) TM = 54°C

PCR 22mars2016 AT1bR

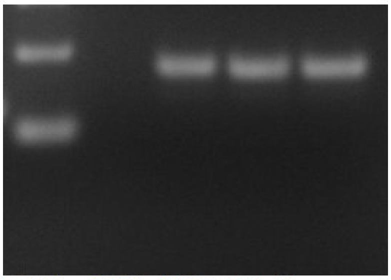

100 bp MM / BS DNA from mouse :
0ng / 50ng / 100ng / 150ng /
Assay AT1bR (167 bp) TM = 56°C

PCR 23 mars 2016 **AT2R**

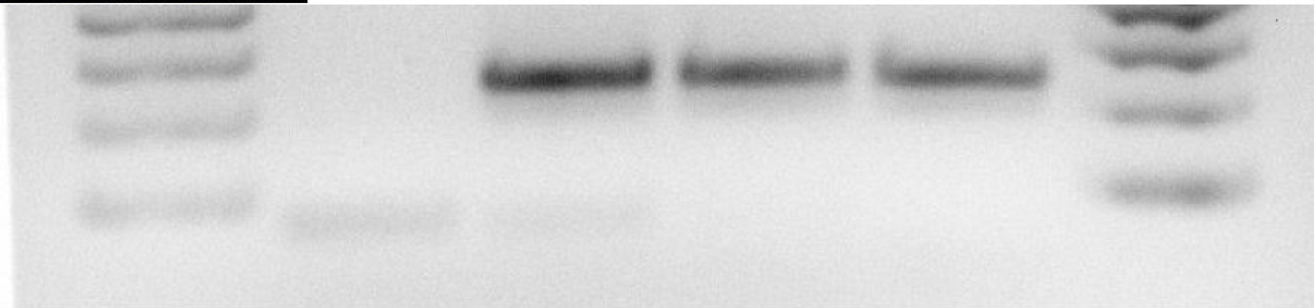

100 bp MM 0 ng 50 100 150 MM
TM = 54 °C Assay **AT2R (245 bp)**

Validation of assay using methylated standards varying between 0 and 100 % methylation.

AT1aR (all sites validated)				
standard	cpg1	cpg2	cpg3	cpg4
0%	1	1	4	2
5%	11	10	18	11
10%	25	19	28	19
25%	37	34	50	36
50%	61	58	80	38
75%	79	77	100	78
100%	87	84	100	98

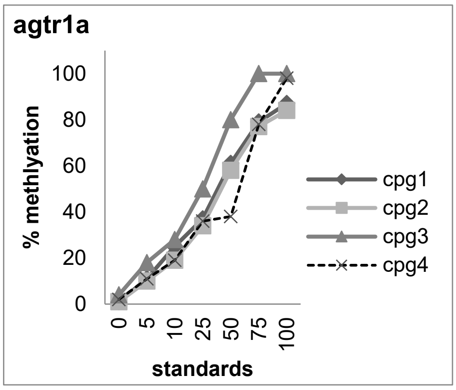

AT1bR (all sites validated)					
standard	cpg1	cpg2	cpg3	cpg4	cpg5
0%	1	0	4	2	ND
5%	4	5	9	4	ND
10%	10	11	17	12	ND
25%	21	26	32	18	ND
50%	41	47	57	35	ND
75%	67	73	81	56	ND
100%	90	94	100	79	91

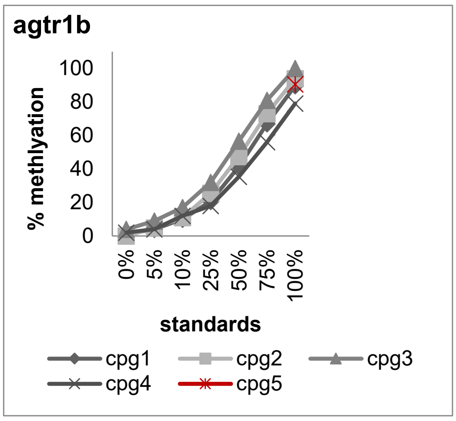

**AT2R assay, CpG1 and CpG2 validated**				
standard	cpg1	cpg2	cpg3	cpg4
0%	6	6	66	6
5%	14	13	47	6
10%	17	12	57	6
25%	39	29	100	7
50%	58	43	100	6
75%	80	58	100	7
100%	96	82	100	18

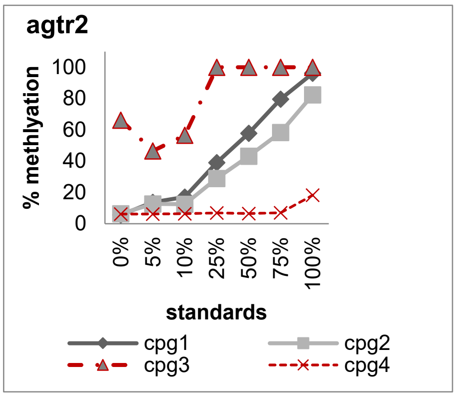

Illustrative pyro-histograms

AT1AR - 0% methylated

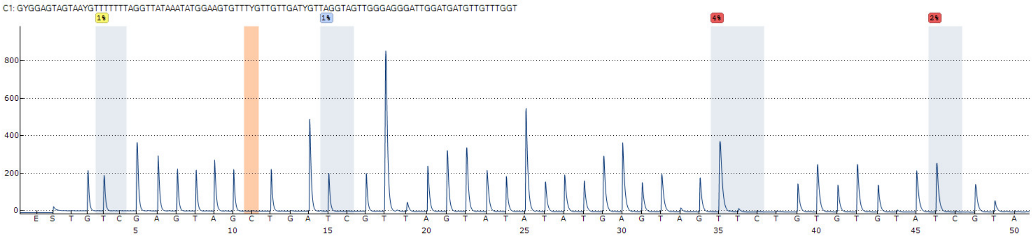

AT1AR - 75% methylated

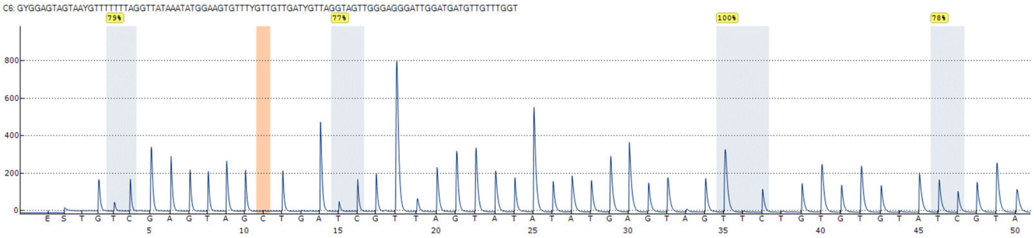

AT1BR - 0% methylated

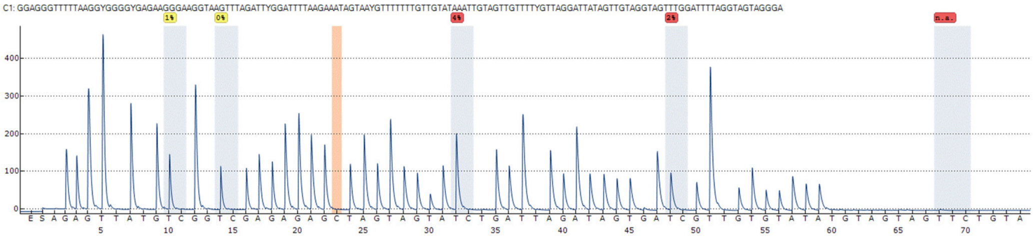

AT1BR - 100% methylated

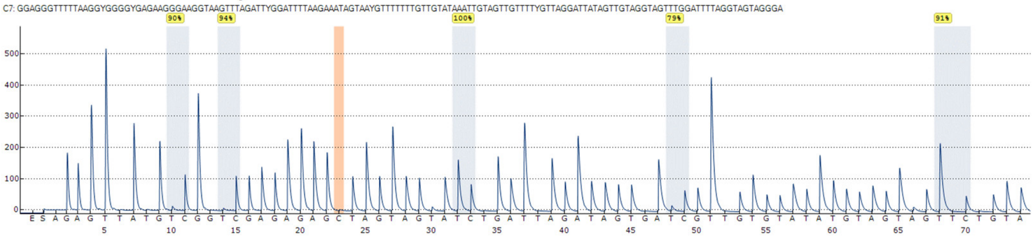

AT2R - 0% methylated

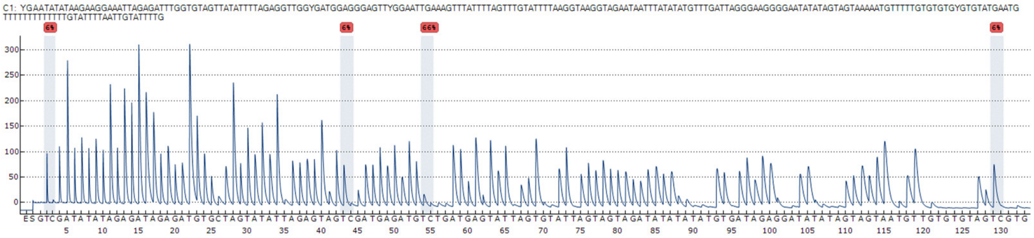

AT2R - 100% methylated

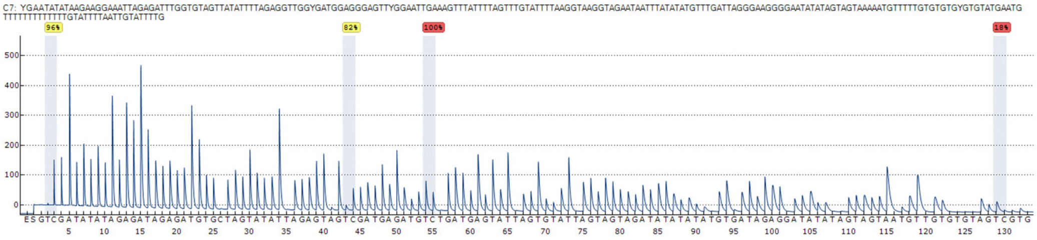

Supplement: Supplementary file 1 [file ijms-23-13357-s001.zip › ijms-1958065 - supplementary.pdf]
